# P02.15. Acupuncture improves *in vitro* fertilization live birth outcomes: a retrospective chart review

**DOI:** 10.1186/1472-6882-12-S1-P71

**Published:** 2012-06-12

**Authors:** L Hullender Rubin, M Opsahl, D Ackerman

**Affiliations:** 1 Oregon College of Oriental Medicine, Research Department, Portland, USA; 2 Northwest Center for Reproductive Medicine, Kirkland, USA

## Purpose

The effectiveness of adjuvant acupuncture on the day of embryo transfer (ET) to improve *in vitro* fertilization (IVF) outcomes is unclear. Most trials thus far have shown some or no effect. In this retrospective chart review, we investigated if a unique acupuncture protocol had any effect on IVF live births.

## Methods

From 2008–2009, patients at a private infertility clinic in Kirkland, WA could elect onsite adjuvant acupuncture on the day of ET. Of 464 patients who received ET, 188 elected acupuncture (Acu) and 276 did not (No Acu). The Acu group received two standardized acupuncture treatments. Prior to ET, the following points were needled: GV20, PC6, ST29, SP8, LV3 and R6 with Shenmen and Brain on the left ear and Uterus and Endocrine on the right ear. Post ET, the same points on opposite ears and LI4, SP10, ST36, and SP6 were needled. Live birth outcomes were analyzed using logistic regression with age and follicle stimulating hormone (FSH) as covariates. Differences across categories of maternal age were evaluated with crude risk ratios.

## Results

There were 106 (56%) live births in the Acu group and 126 (35%) in the No Acu group (OR=1.68, CI=1.14-2.46, p<0.001). Live births associated with acupuncture were significantly higher in the 35-37 age group (RR=1.86, CI=1.18–2.94, p=0.01) and the 38-40 age group (RR=1.79, CI=1.02–3.15, p=0.04). In the under 35 and over 40 age groups, there were somewhat more live births in the Acu groups although the differences did not reach statistical significance.

## Conclusion

IVF live birth outcomes may be improved with this unique acupuncture protocol. This finding should be taken cautiously as more rigorous research is needed.

